# Comparison of hemodynamic, biochemical and hematological parameters of healthy pregnant women in the third trimester of pregnancy and the active labor phase

**DOI:** 10.1186/1471-2393-11-33

**Published:** 2011-05-06

**Authors:** Fernando Rodríguez-Dennen, Joel Martínez-Ocaña, Simón Kawa-Karasik, Luis Villanueva-Egan, Norberto Reyes-Paredes, Ana Flisser, Angélica Olivo-Díaz

**Affiliations:** 1 Hospital General "Dr. Manuel Gea González", Calzada de Tlalpan 4800, México 14080 DF, México; 2 Instituto de Oftalmología Fundación Conde de Valenciana, Chimalpopoca 14 Colonia Obrera, C.P. 06800 DF, México; 3 Hospital de la Mujer, Prol. Salvador Díaz Mirón 374, México 11340 DF, México; 4 Facultad de Medicina, Universidad Nacional Autónoma de México, México 04510 DF, México

## Abstract

**Background:**

Pregnancy is accompanied by several hemodynamic, biochemical and hematological changes which revert to normal values after labor. The mean values of these parameters have been reported for developed countries, but not for Mexican women. Furthermore, labor constitutes a stress situation, in which these factors may be altered. It is known that serologic increase of heat shock protein (Hsp) 70 is associated with abnormal pregnancies, presenting very low level in normal pregnant women. Nevertheless, there are no studies where these measurements are compared in healthy pregnant women at their third trimester of pregnancy (3TP) and the active labor phase (ActLP).

**Methods:**

Seventy five healthy Mexican pregnant women were included. Hemodynamic, biochemical and hematological parameters were obtained in all cases, and serum Hsp70 levels were measured in a sample of 15 women at 3TP and at ActLP.

**Results:**

Significant differences were found in most analysis performed and in Hsp70 concentration at 3TP as compared to ActLP, however all were within normal range in both conditions, supporting that only in pathological pregnancies Hsp70 is drastically increased.

**Conclusion:**

Results obtained indicate that 3TP and ActLP have clinical similarities in normal pregnancies, therefore if abnormalities are found during 3TP, precautions should be taken before ActLP.

## Background

During pregnancy several hemodynamic, biochemical and hematological modifications occur as part of the physiological adaptation of the body to this condition. For instance, maternal blood pressure (BP) initially decreases at 8 weeks of gestation or earlier, the decrease in diastolic BP is higher than that in systolic BP [1]. The diastolic BP has the lowest value at midpregnancy and returns to prepregnancy levels by term; in most studies it rarely exceeds prepregnancy or postpartum values. However, some investigators have reported that at term and in the third trimester, BP is higher than in matched nonpregnant controls [2,3]. In contrast, several respiratory parameters do remain essentially unchanged during pregnancy, such as total lung capacity, vital capacity, lung compliance and diffusion capacity. Respiratory rate (RR) does not change also during pregnancy and tachypnea with greater than 20 breaths per minute should be considered abnormal in the pregnant woman [4,5]. Nonetheless, minute ventilation, tidal volume and oxygen consumption increase 20% to 50%, whereas functional residual capacity raise only 20%, total lung capacity decreases by about 4% to 5%, mostly caused by the upward displacement of the diaphragm, increased metabolic rate, changes in the mechanics of breathing, and increases in progesterone level [5,6]. Additionally, oxygen consumption increases by 30% to 60% during the course of pregnancy and maternal arterial partial pressure of CO_2 _decreases to a level of 26 to 32 mm Hg as a result of increased minute ventilation [4-7]. With respect to blood analysis, only slight changes in the amount of different white cells, platelets, hemoglobin and creatinine have been described [8,9]. The mean of hemodynamic, such as BP, heart rate (HR), respiratory rate (RR); biochemical, as blood urea nitrogen (BUN), creatinine, hemoglobin, hematocrite, and glucose; and cellular reference values have been adopted from textbooks that mainly refer to Caucasian subjects [10,11]; few studies report hemodynamic [12,13], biochemical [9,14], and hematological [15,16] values for female living in developing countries, being scantly or absent for Mexican women.

In addition, since labor constitutes a stress situation, therefore, heat shock proteins (Hsps), particularly Hsp70, could be increased in serum in the active labor phase (ActLP). Hsps represent 2-15% of total cell proteins, being their main function preventing inadequate activity inside the cell (apoptosis, non regulated inflammation, abnormal protein degradation, abnormal metabolite production, etc,) [17]. As part of their homeostatic response, stress proteins are fundamental in the adaptive responses of unicellular and multicellular organisms. They are implicated in a great variety of phenomena, including immune response modulation, hyperthermia, hyperoxia, ischemia and other alterations [17,18]. Elevated serum Hsps levels are associated with various physiopathological situations during pregnancy and Hsp70 is consistently expressed in normal female reproductive tissues during pregnancy [19-21]. Women with preterm delivery and preeclampsia have higher Hsp70 concentrations (mean ± Standard error of the mean) (35.3 ± 9.6 and 24.4 ± 3.6 ng/mL, respectively), as compared to normal pregnant (6.1 ± 0.6 ng/mL) and non pregnant (2.4 ± 0.6 ng/mL) women [22-24]. It has been also found elevated Hsp70 levels in transient hypertension of pregnancy, preeclampsia and superimposed preeclampsia (median (25-75 percentile) 0.66 (0.52-0.84), 0.55 (0.42-0.80) and 0.61 (0.42-0.91) ng/mL respectively) [25,26] as well as in pregnant asthmatics (0.44 (0.36-0.53) ng/mL), compared to healthy pregnant women (0.21 (0-0.27) ng/mL). Fetal birth weight of asthmatic mothers was significantly smaller than of healthy controls, but in the normal range (3,230 g (2,690-3,550) versus 3,550 g (3,450-3,775) [27]. In preeclampsia, increased serum Hsp70 levels reflect systemic inflammation, oxidative stress and hepatocellular injury [28]. Moreover, serum Hsp70 levels are significantly higher in patients with the syndrome of hemolysis, elevated liver enzymes, and low platelet count (HELLP syndrome, 2.02 (0.76-2.23) ng/mL) than in severely preeclamptic patients without it (0.54 (0.47-0.79) ng/mL). In HELLP syndrome, elevated serum Hsp70 level indicates tissue damage (hemolysis and hepatocellular injury) and disease severity [29,30]. However, circulating levels of anti-Hsp antibodies are not altered in preeclampsia [31]. Recently, significantly lower serum Hsp70 levels in healthy pregnant women than in healthy non-pregnant women were described; also a statistically significant negative correlation between maternal age and serum Hsp70 concentration and a significant positive correlation between gestational age and serum Hsp70 level in healthy pregnant women were found [32].

## Methods

Seventy five healthy pregnant women with uncomplicated pregnancies (aged 16-35 years) were included. Two measurements were performed, one at their third trimester of pregnancy (3TP) and the other one at active labor phase (ActLP), being each woman its own control. ActLP was considered when cervix dilatation was bigger than 8 cm, frequency of uterine contractions higher than 2 each 10 min and duration of contraction more than 30 sec. Women were recruited from the Division of Obstetrics in the Hospital General "Dr. Manuel Gea Gonzalez". The study was approved by the Ethics and Investigation Committees of the same hospital. After explaining the purpose of the study to each pregnant woman, written consent was obtained.

A complete medical history, values for BP, HR, RR and fetal heart rate (FHR) (hemodynamic variables), BUN, creatinine, hemoglobin, hematocrite and glucose (biochemical variables), leucocytes, neutrophils, lymphocytes, monocytes, eosinophils, basophils and platelets (hematological variables), were obtained at 3TP and ActPL from all women enrolled. Women did not have any active disease such as hypertension, gynecological complications of pregnancy or other acute or chronic diseases. If any complication in pregnancy, birth or immediately after birth arouse, the woman was excluded from the study.

A sample of 15 mL of peripheral blood was obtained for biochemical, hematological and Hsp70 measurement during the 3TP in the morning after overnight fasting; and in the ActLP. Hsp70 serum levels were measured only in a randomly selected sample of the women, corresponding to 20% of the studied population (n = 15). Hsp70 specific ELISA kit (StressGen Biotechnologies Corp, Victoria, Canada) was used, with 100 μl of a 1:5 dilution of each serum, in duplicate wells in the Hsp70 plate, following instructions of the manufacturer. Absorbance values were obtained at 450 nm. This assay has a minimum sensitivity of 0.2 ng/mL. Statistical analysis was performed by paired T student test. Statistical significance was considered at *p *value ≤ 0.05.

## Results

Mean values ± standard deviation (SD) of the hemodynamic, biochemical and hematological variables studied in the pregnant women at 3TP and at ActPL are shown in Table 1. When correlating these parameters in the two stages, a significant difference in most values was found, with exception of basophils that showed no change.

**Table 1:** Average values of hemodynamic variables and blood analysis of 75 women at 3TP and ActLP.

	3TP*	ActLP*	*p*
**Hemodynamic variables**			
BP Diastolic (mmHg)	64 ± 7	82 ± 5	<0.0001
BP Systolic (mmHg)	103 ± 9	129 ± 7	<0.0001
HR (bpm)	79 ± 9	94 ± 8	<0.0001
RR (rpm)	19 ± 3	30.5 ± 5	<0.0001
FHR (bpm)	141.7 ± 4	153 ± 3	<0.0001
Temperature°C	36.2 ± 0.28	36.7 ± 0.19	<0.0001
**Biochemical and celular variables**			
Leucocytes (×10^3^)	8.3 ± 1.4	10.3 ± 1.4	<0.0001
Neutrophils (%)	65.6 ± 9.0	77.7 ± 7.9	<0.001
Lymphocytes (%)	26.6 ± 7.8	16.6 ± 7.0	<0.001
Monocytes (%)	6.1 ± 1.9	4.8 ± 1.4	<0.01
Eosinophils (%)	1.2 ± 0.9	0.4 ± 0.3	<0.01
Basophils (%)	0.4 ± 0.2	0.3 ± 0.3	NS
Platelets (×10^3^)	267.0 ± 51.0	216.0 ± 60.0	<0.01
BUN (mg/dL)	6.8 ± 1.6	8.4 ± 1.9	<0.05
Creatinine (mg/dL)	0.6 ± 0.09	0.8 ± 0.12	<0.001
Hemoglobin (gr/dL)	13.2 ± 1.4	12.0 ± 1.1	<0.001
Hematocrite (%)	39 ± 3.8	36.3 ± 3.1	<0.001
Glucose (mg/dL)	84 ± 10	95 ± 8	<0.001

BP: blood pressure, HR: heart rate, RR: respiratory rate, FHR: fetal heart rate, BUN: blood urea nitrogen. SD: standard deviation, 3TP: third trimester of pregnancy, ActLP: active labor phase, bpm: beats per minute, rpm: respirations per minute, NS: not significant. *Values are mean ± SD.

**Figure 1:**
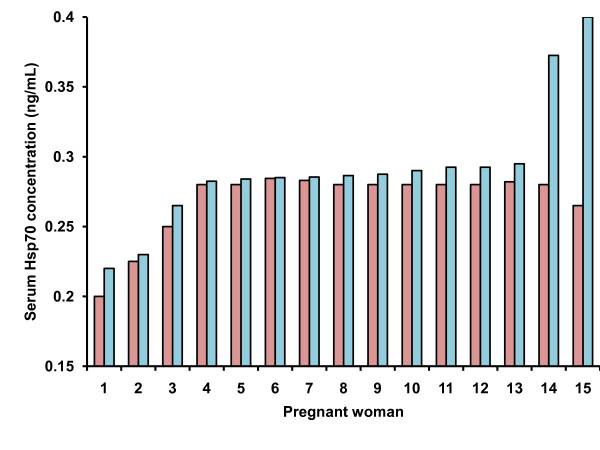
**Concentration of HSP70 in serum**. Concentration of HSP70 in serum of women using ELISA at the third trimester of pregnancy (3TP, pink bars) and the same women at the active labor phase (ActLP, blue bars).

Figure 1 shows the serum concentration of Hsp70 at 3TP and ActLP. The mean (±SD) was 0.268 ± 0.02 ng/mL in 3TP and 0.291 ± 0.04 ng/mL in ActLP. The difference was statistically significant (p = 0.03) as it would be expected because of the physiological nature of stress presented in the ActLP. Even so all values were within normal concentrations and similar to those previously reported for healthy pregnant women [33]. Only two women showed a higher difference between the two stages, which could indicate some complication during ActLP, but the values were still within normal concentrations. Mean (±SD) of gestational week, age, and weight of newborn were 38.3 ± 0.8, 24.3 ± 6.4, and 2,987 ± 306.2 g, respectively in the women included for Hsp70 quantification.

## Discussion

Differences in the hemodynamic, biochemical and hematological variables indicate a response to a physiological stress during the ActLP, showing a higher significance in hemodynamic variables. No data have been described with respect to Hsp70 in Mexican healthy pregnant women. Statistical significance was found in Hsp70 amounts in both stages, but still in normal ranges. Even though decidual stromal, cytotrophoblast, and syncytiotrophoblast cells show a constant production of Hsp70 during the whole pregnancy [20], this protein is not normally released but only in pathological conditions, such as preeclampsia, preterm birth, superimposed preeclampsia, HELLP syndrome, and pregnant asthmatics, in which an important increase in serum Hsp70 concentration (up to 100 times the initial value) has been found [22,25-31]. Besides, Hsp70 shows important differences in healthy individuals in several instances: its serum concentration is inversely proportional to age [24], but directly proportional to the amount of exercise [34]. Our results indicate that in healthy pregnant women the levels of Hsp70 are in normal range, therefore higher levels may be indicative of a pathological condition in pregnancy. Thus, measure of Hsp70 at 3TP might be useful as predictive test of complicate pregnancies. No correlation between Hsp70 level and neonatal birth weight was found, even in the two women where the amount of Hsp70 at ActLP was higher than in the others. Neonatal birth weight was within the normal range (3,057 g and 3,426 g). The study is limited because, due to technical reasons, Hsp70 serum levels were measured only in 20% of the studied population (n = 15) and the weight of the remaining 60 newborns was not available.

It is outstanding that in a literature review, hemodynamic, biochemical and hematological parameters of pregnant women have only been reported in very few countries (Israel [8], Australia [11], Nigeria [12], United States of America [9,13], and Venezuela [14]), and reference values for healthy nonpregnant and pregnant women reported in obstetric books are from the USA [10]; only recently Ethiopia and Uganda are defining reference values [15,16]. More information would be valuable in order to define if ethnical, geographical and other variables modify these reference values in pregnant and nonpregnant women.

## Conclusions

This is the first study that provides hemodynamic, biochemical and hematological parameters as well as Hsp70 measurements in normal Mexican women during the third trimester of pregnancy and the active labor phase. Most values showed significant differences, nonetheless, all are among the established normal values. Results obtained indicate that 3TP and ActLP have clinical similarities in normal pregnancies, therefore if abnormalities are found during 3TP, precautions should be taken before ActLP.

## List of abbreviations used

3TP: Third trimester of pregnancy; ActLP: Active labor phase; BP: Blood pressure; BUN: Blood urea nitrogen; FHR: Fetal heart rate; HR: Heart rate; Hsp70: Heat Shock Protein 70; NA: Not available; NS: Not significant; RR: Respiratory rate; SD: Standard deviation;

## Competing interests

The authors declare that they have no competing interests.

## Authors' contributions

FRD and JMO collected and processed samples, performed HSP70 ELISA and statistical analyses. LVE and NRP participated in the analysis of the hemodynamic and blood parameters. SKK contributed to data analysis and in the revision of the manuscript. AOD and AF designed the study and wrote the manuscript. All authors read and approved the final manuscript.

## Pre-publication history

The pre-publication history for this paper can be accessed here:

http://www.biomedcentral.com/1471-2393/11/33/prepub

## References

[B1] Hunter S Robson SC Adaptation of the maternal heart in pregnancy Br Heart J 1992 68 540 543 10.1136/hrt.68.12.540 1467047 PMC1025680

[B2] Duvekot JJ Peeters LL Maternal cardiovascular hemodynamic adaptation to pregnancy Obstet Gynecol Surv 1994 49 S1 S14 10.1097/00006254-199412011-00001 7877788

[B3] Clapp JF Capeless E Cardiovascular function before, during and after the first and subsequent pregnancies Am J Cardiol 1997 80 1469 1473 10.1016/S0002-9149(97)00738-8 9399724

[B4] Wise RA Polito AJ Krishnan V Respiratory physiologic changes in pregnancy Immunol Allergy Clin North Am 2006 26 1 12 10.1016/j.iac.2005.10.004 16443140

[B5] Bobrowski RA Pulmonary physiology in pregnancy Clin Obstet Gynecol 2010 53 285 300 10.1097/GRF.0b013e3181e04776 20436304

[B6] Chesnutt AN Physiology of normal pregnancy Crit Care Clin 2004 20 609 615 10.1016/j.ccc.2004.06.001 15388191

[B7] Hill CC Pickinpaugh J Physiologic changes in pregnancy Surg Clin North Am 2008 88 391 401 10.1016/j.suc.2007.12.005 18381119

[B8] Efrati P Presentey B Margalith M Rozenszajn L Leukocytes of normal pregnant women Obstet Gynecol 1964 23 429 432 14128474

[B9] Knight EM Spurlock BG Edwards CH Johnson AA Oyemade UJ Cole OJ West WL Manning M James H Laryea H Westney OE Jones S Westney LS Biochemical profile of African American women during three trimesters of pregnancy and at delivery J Nutr 1994 124 943S 953S 8201445 10.1093/jn/124.suppl_6.943S

[B10] Gabbe SG Obstetrics: Normal and Problem Pregnancies 2007 New York: Churchill Livingstone, Elsevier

[B11] Simmons LA Gillin AG Jeremy RW Structural and functional changes in left ventricle during normotensive and preeclamptic pregnancy Am J Physiol Heart Circ Physiol 2002 283 H1627 H1633 12234817 10.1152/ajpheart.00966.2001

[B12] Gaya B Talatu G Adelaiye A Cardiopulmonary changes in pregnant women in Sabon-Gari local government area, of Kaduna state, Nigeria The Internet Journal of Health 2009 9 2 ISSN: 1528-8315

[B13] Mesa A Jessurun C Hernandez A Adam K Brown D Vaughn WK Wilansky S Left ventricular diastolic function in normal human pregnancy Circulation 1999 99 511 517 9927397 10.1161/01.cir.99.4.511

[B14] Rached de Paol II Azuaje Sanchez A Henriquez Perez G Cambios en las variables hematológicas y bioquímicas durante la gestación en mujeres eutróficas An Venez Nutr 2002 15 11 17 ISSN 0798-0752 12012560

[B15] Tsegaye A Messele T Tilahun T Hailu E Sahlu T Doorly R Fontanet AL Rinke de Wit TF Immunohematological reference ranges for adult Ethiopians Clin Diagn Lab Immunol 1999 6 410 414 10225845 10.1128/cdli.6.3.410-414.1999 PMC103732

[B16] Lugada ES Mermin J Kaharuza F Ulvestad E Were W Langeland N Asjo B Malamba S Downing R Population-based hematologic and immunologic reference values for a healthy Ugandan population Clin Diagn Lab Immunol 2004 11 29 34 14715541 10.1128/CDLI.11.1.29-34.2004 PMC321349

[B17] Ellis RJ van der Vies SM Molecular chaperones Annu Rev Biochem 1991 60 321 347 10.1146/annurev.bi.60.070191.001541 1679318

[B18] Moseley P Stress Proteins and the immune response Immunopharmacology 2000 48 299 302 10.1016/S0162-3109(00)00227-7 10960671

[B19] Shah M Stanek J Handwerger S Differential localization of heat shock proteins 90, 70, 60 and 27 in human decidua and placenta during pregnancy Histochem J 1998 30 509 518 10.1023/A:1003259907014 10192534

[B20] Ziegert M Witkin SS Sziller I Alexander H Brylla E Härtig W Heat shock proteins and heat shock protein-antibody complexes in placental tissues Infect Dis Obstet Gynecol 1999 7 180 185 10449265 10.1002/(SICI)1098-0997(1999)7:4<180::AID-IDOG3>3.0.CO;2-7 PMC1784741

[B21] Sotiriou S Liatsos K Ladopoulos I Arvanitis DL A comparison in concentration of heat shock proteins (HSP) 70 and 90 on chorionic villi of human placenta in normal pregnancies and in missed miscarriages Clin Exp Obstet Gynecol 2004 31 185 190 15491060

[B22] Fukushima A Kawahara H Isurugi C Syoji T Oyama R Sugiyama T Horiuchi S Changes in serum levels of heat shock protein 70 in preterm delivery and pre-eclampsia J Obstet Gynaecol Res 2005 31 72 77 10.1111/j.1447-0756.2005.00244.x 15669997

[B23] Pockley AG Shepherd J Corton JM Detection of heat shock protein 70 (HSP70) and anti Hsp70 antibodies in the serum of normal individuals Immunol Invest 1998 27 367 377 10.3109/08820139809022710 9845422

[B24] Jin X Wang R Xiao C Cheng L Wang F Yang L Feng T Chen M Chen S Fu X Deng J Wang R Tang F Wei Q Tanguay RM Wu T Serum and lymphocyte levels of Heat Shock protein 70 in aging: a study in the normal Chinese population Cell Stress Chaperones 2004 9 69 75 15270079 10.1379/477.1 PMC1065308

[B25] Molvarec A Tamási L Losonczy G Madách K Prohászka Z Rigó J Jr Circulating heat shock protein 70 (HSPA1A) in normal and pathological pregnancies Cell Stress Chaperones 2010 15 237 247 10.1007/s12192-009-0146-5 19821156 PMC2866993

[B26] Molvarec A Prohászka Z Nagy B Szalay J Füst G Karádi I Rigó J Jr Association of elevated serum heat-shock protein 70 concentration with transient hypertension of pregnancy, preeclampsia and superimposed preeclampsia: a case-control study J Hum Hypertens 2006 20 780 786 10.1038/sj.jhh.1002060 16761027

[B27] Tamási L Bohács A Tamási V Stenczer B Prohászka Z Rigó J Jr Losonczy G Molvarec A Increased circulating heat shock protein 70 levels in pregnant asthmatics Cell Stress Chaperones 2010 15 295 300 10.1007/s12192-009-0143-8 19777374 PMC2866990

[B28] Molvarec A Rigó J Jr Lázár L Balogh K Makó V Cervenak L Mézes M Prohászka Z Increased serum heat-shock protein 70 levels reflect systemic inflammation, oxidative stress and hepatocellular injury in preeclampsia Cell Stress Chaperones 2009 14 151 159 10.1007/s12192-008-0067-8 18686014 PMC2727991

[B29] Molvarec A Prohászka Z Nagy B Kalabay L Szalay J Füst G Karádi I Rigó J Jr Association of increased serum heat shock protein 70 and C-reactive protein concentrations and decreased serum alpha(2)-HS glycoprotein concentration with the syndrome of hemolysis, elevated liver enzymes, and low platelet count J Reprod Immunol 2007 73 172 179 10.1016/j.jri.2006.07.002 17023052

[B30] Madách K Molvarec A Rigó J Jr Nagy B Pénzes I Karádi I Prohászka Z Elevated serum 70 kDa heat shock protein level reflects tissue damage and disease severity in the syndrome of hemolysis, elevated liver enzymes, and low platelet count Eur J Obstet Gynecol Reprod Biol 2008 139 133 138 10.1016/j.ejogrb.2007.12.012 18249485

[B31] Molvarec A Derzsy Z Kocsis J Boze T Nagy B Balogh K Makó V Cervenak L Mézes M Karádi I Prohászka Z Rigó J Jr Circulating anti-heat-shock-protein antibodies in normal pregnancy and preeclampsia Cell Stress Chaperones 2009 14 491 498 10.1007/s12192-009-0102-4 19205928 PMC2728282

[B32] Molvarec A Rigó J Jr Nagy B Walentin S Szalay J Füst G Karádi I Prohászka Z Serum heat shock protein 70 levels are decreased in normal human pregnancy J Reproductive Immunol 2007 74 163 169 10.1016/j.jri.2006.12.002 17296233

[B33] Livingston JC Ahokas R Haddad B Sibai BM Awaads R Heat Shock protein 70 is not increased in women with severe preeclampsia Hypertens Pregnancy 2002 21 123 126 10.1081/PRG-120004767 12175440

[B34] Fehrenbach E Niess AM Voelker K Northoff H Mooren FC Exercise intensity and duration affect blood soluble HSP72 Int J Sports Med 2005 26 552 557 10.1055/s-2004-830334 16195988

